# Editorial: Traditional medicine and chronic inflammatory diseases

**DOI:** 10.3389/fphar.2023.1202976

**Published:** 2023-04-27

**Authors:** Wei Liu, Yang-Cheng Liu, Zheng Xiang

**Affiliations:** School of Pharmaceutical Science, Liaoning University, Shenyang, China

**Keywords:** traditional medicine, chronic inflammatory diseases, nature product, liver injury, enteritis, arthritis, diabetes nephropathy

Inflammatory response is the most common pathological reaction in the human body. If encountering infection and injury, the production of inflammation in the body is usually localized, transient, and belongs to acute inflammation. If inflammatory factors persist and damage tissues, acute inflammation will gradually transform into chronic inflammation. In addition, chronic inflammation can also occur due to the influence of social environment and lifestyle (Furman et al., 2019). Cardiovascular disease, cancer, diabetes, chronic nephritis, nonalcoholic fatty liver disease, autoimmune disease and degenerative disease based on the chronic inflammation seriously threaten human life and health (Straub, 2017; Yang et al., 2020). Since the development of science, people have gradually realized the important harm of chronic inflammation to body tissues. The characteristic of chronic inflammation is that there are often no obvious symptoms between episodes, such as chronic cholecystitis (Wang et al., 2021), chronic pyelonephritis (Tsuji et al., 2007), *etc.* Chronic inflammation can also occur slowly and insidiously, with no clinical manifestations of acute inflammation. It is common in intracellular viral infections, where the virulence of these pathogens is not strong, but they can cause immune responses. Long term exposure to non-degradable but potentially toxic substances or the effects of persistent autoimmune diseases can also lead to the occurrence of chronic inflammation.

The multiple causes of chronic inflammation lead to a higher incidence in the human body, and because its treatment cycle is longer than acute inflammation, it is crucial to reduce the harmful effects on the human body during the treatment process. Compared to chemical drugs, traditional medical treatment methods have lower side effects. The role of natural products from medicinal plants, medicinal animals, and functional foods in disease treatment is increasingly valued, and the therapeutic effects and mechanisms of traditional medicine on diseases have become a research hotspot. Therefore, the potential of traditional medicine for treating chronic inflammatory diseases needs to be developed. This Research Topic selects traditional medicine and chronic inflammatory diseases as the themes, committed to promoting more meaningful research on the treatment of chronic inflammatory diseases through traditional medicine, exploring more efficient and less side effects treatment methods for chronic inflammatory and improve the relevant theoretical foundation research.

In this Research Topic, a total of 15 articles were published, including two review articles and 13 original research articles. These articles focus on the therapeutic relationships between various traditional medicine drugs and inflammatory diseases, and conduct in-depth research through various research methods such as metabolomics and microbic genomics. Two review articles focus on the effects of two traditional drugs from plant and animal, respectively, on various inflammations. Wang et al. reviewed the research findings of *Asparagus cochinchinensis* (Lour.) Merr and the progress in botany, traditional use, phytochemistry, pharmacology and application were summarized. Lee et al. reviewed the positive effects of Channa striatus on inflammatory conditions such as gastric ulcers, dermatitis, osteoarthritis, and allergic rhinitis. Compared to the widely used non-steroidal anti-inflammatory drugs, the natural ingredient extracted from Channa striatus is more user-friendly and can reduce the risk of peptic ulcers, acute renal failure, stroke, and myocardial infarction caused by the use of chemical drugs such as non-steroidal anti-inflammatory drugs.

Three articles focus on inflammation related to liver injury and liver cancer. Due to changes and advancements in lifestyle, liver injury is a very common disease in modern society. Wang et al. revealed the material basis of *Liquidambaris fructus* extract in treating liver cancer cells and demonstrated its effects on inhibiting tumor cell growth, promoting tumor cell apoptosis, reducing inflammatory response, protecting liver cells, and improving the survival status of tumor rats through metabolomics methods. Pathway analysis indicates that the above effects are related to key signaling pathways such as PTEN/PI3K/Akt and fatty acid metabolism. Qiao et al. found that *Patrinia villosa* (Thunb.) Juss has a therapeutic effect on liver injury caused by inflammation, reducing liver tissue structural damage, cytoplasmic vacuolization, cell swelling, and inflammatory cell infiltration. Its therapeutic effect is related to the metabolism of alanine, aspartic acid, and glutamate, as well as the TCA cycle pathway. The research results of Jin et al. suggested that quercetin 7-rhamnoside can treat cholestatic hepatitis by regulating bile acid secretion and reducing inflammation. Two articles focus on skin related inflammatory diseases. The data of Wang et al. indicated that, β-elemene can reduce the expression of inflammatory cytokines secreted by M1-Mø and induce apoptosis of psoriasis keratinocytes to suppress inflammation, thereby significantly alleviating the symptoms of the psoriasis mouse model; Han et al. found that oxymatrine can improve skin inflammation symptoms in atopic dermatitis mice by upregulating the expression of cytokine signaling inhibitor 1, inhibiting the activation of JAK-STAT3 pathway, and blocking the activation of T lymphocytes. In addition, two articles focus on traditional medical treatment methods for arthritis; Jiao et al. used the extraction of *Cordyceps militaris* (Linn.) Link to treat gouty arthritis and achieved good results. The extract of cordyceps militaris significantly alleviated the inflammatory progression of gouty arthritis and ameliorated the onset of gouty arthritis. The underlying mechanism may be related to triggering the cytokine-cytokine receptor interaction signaling pathway to inhibit the activation of the inflammasome and regulate the immune system. And it regulates the inflammatory response induced by monosodium urate crystals through the genes CCL7, CSF2RB, and IL-1β. Han et al. used *X. mongolicum* Kitag to treat rheumatoid arthritis, another type of chronic arthritis. The main active ingredient of *X. mongolicum* in the treatment of rheumatoid arthritis is sesquiterpene lactone. Sesquiterpen lactones enriched fraction from *X. mongolicum* may pass NF-κB and MAPK signaling pathways inhibit M1 macrophage polarization, thereby exerting their therapeutic effect on rheumatoid arthritis. The inflammatory response in the brain is a more challenging area of onset and is more difficult to effectively treat. Qiao et al. found that Radix Ginseng (the dry root and rhizome of *Panax ginseng* C. A. Meyer) and Semen Ziziphi Spinosae (the dry and mature seed of *Ziziphus jujuba* Mill.var.*spinosa* (Bunge) HuexH.F.Chou) drug pair can improve sleep in insomnia rats by regulating the GLU/GABA-GLN metabolic cycle and gut microbiota. Yan et al. proved that water extract of *Broussonetia Papyrifera* (L.) L'Hér. ex Vent. fruits can enhance the proliferation of neural stem cells and improve neurogenesis, so as to effectively repair the damaged neurons in the hippocampus of APP/PS1 transgenic mice and improve cognitive impairment. Its function is related to the activation of Wnt/β-catenin signaling pathway. Yang et al. proved that Bufei Jianpi granule, which is a traditional Chinese medicine formulation including *Astragalus mongholicus* Bunge [Fabaceae; *A. mongholicus* radix], *Codonopsis pilosula* (Franch.) Nannf [Campanulaceae; *C. pilosula* radix], *Polygonatum kingianum* Collett & Hemsl [Asparagaceae; *P. kingianum* rhizoma], *Atractylodes macrocephala* Koidz [Asteraceae; *Atractylodis macrocephalae* rhizoma], *Poria cocos* (Schw.) Wolf [Poromycelidae; *Poria*], *Fritillaria thunbergii* Miq [Liliaceae; *Fritillariae thunbergii* bulbus], *Pheretima aspergillum* (E. Perrier) [Lumbricidae; *Pheretima*], *Magnolia officinalis* Rehder & E. H. Wilson [Magnoliaceae; *Magnoliae officinalis* cortex], *Citrus reticulata* Blanco [Rutaceae; *Citri reticulatae* pericarpium], *Aster tataricus* L. f [Asteraceae; *Asteris radix* et rhizoma], *Epimedium brevicornu* Maxim [Berberidaceae; *Epimedii* folium], and *Ardisia japonica* (Thunb.) Blume [Primulaceae; *Ardisiae japonicae* herba] at a ratio of 12:6:12:9:9:6:9:6:9:6:6:15, has a pharmacological effect in the treatment of chronic obstructive pulmonary disease, and downregulate the levels of IL-6, IL-8, TNF-α, PGE2, MMP-9, and NO in the serum and bronchoalveolar lavage fluid. Wang et al. focus on the intestinal inflammatory disease. It was been found that *K. indica* (L.) Sch Bip could significantly reduce the atypical hyperplasia in colon tissue, and inhibit the expression of proinflammatory factors such as IL-6, TNF, IL-11, IL-7, *etc. Kalimeris indica* could also restore the level of miR-31–5p in mice, and therefore the downstream LATS2 to inhibit the development of colitis-associated colorectal cancer. Ju et al. found that *Cornus officinalis* Torr. ex Dur. may improve the renal injury of diabetic nephropathy rats by blocking the activation of Wnt/β-catenin signaling pathway, regulating the structural composition of intestinal microorganisms, and ultimately playing a role in renal protection. Xu et al. investigated the inhibitory effect of main phenolic acid components of *J. cannabifolia* (Less.) on inflammation caused by PM2.5. P-hydroxybenzoic acid and p-hydroxyphenylacetic acid from *J. cannabifolia* could attenuated inflammation caused by PM2.5 through suppressing TLRs related signal pathway.

Overall, this Research Topic has received widespread attention and published two review articles jointly written by 11 scientists and 13 original research articles jointly designed and written by 93 scientists. This indicates that the treatment of chronic inflammatory diseases through traditional medicine has attracted more and more researchers’ attention, and its advantages over chemical drug methods are becoming increasingly evident. The articles in this Research Topic cover a variety of inflammatory diseases, including liver injury, skin inflammation, arthritis, brain inflammation, obstructive pulmonary disease, enteritis, and diabetes nephropathy, which expand the research field of traditional medicine in the treatment of inflammatory diseases, and indicate the potential of traditional medicine in the treatment of inflammatory diseases. Developing more mature and effective traditional medical treatment methods for chronic inflammatory diseases is an important research direction that can benefit many patients. Better treatment effects and lower toxic effect and side effect will be an important contribution of traditional medicine to the field of chronic inflammatory diseases.
